# Brief Comment on “The Clinical Utility of 3D Electroanatomical Mapping for Atrial Fibrillation Ablation by Pulsed Field Ablation”

**DOI:** 10.1002/joa3.70295

**Published:** 2026-02-12

**Authors:** Sidra Afzal, Nimra Afzal

**Affiliations:** ^1^ Fatima Jinnah Medical University Lahore Pakistan; ^2^ Anglia Ruskin University Cambridge UK

Dear Editor,

I read with keen interest the recent article by Kerley and Keane on the integration of 3D electroanatomical mapping (EAM) with pulsed field ablation (PFA) for atrial fibrillation (AF) [1]. This study gives significant insights into procedure‐related feasibility and the prospective benefit of integrating advanced 3D mapping with newly evolving non‐thermal cardiac ablation techniques.

As appreciated by the authors, this observational study without a control group has limiting interpretation concerning the size of the sample, generalizability, and follow‐up. Despite that, various aspects require further analysis for clinical application and prospective research:

1. **Efficacy comparison**: While acute clinical results are promising, supporting context comparing EAM‐guided PFA with cryoballoon ablation and traditional radiofrequency could elaborate clinical procedural efficiency, fluoroscopy application, and arrhythmia‐free survival among a wider population [2].
2. **Risk assessment**: The short‐term safety evaluation is favorable. Despite that, a comprehensive assessment of cerebrovascular events, atrial tissue remodeling, or esophageal perforation was not incorporated. Recent studies demonstrated that 3D EAM improves the efficiency and accuracy of the procedure in PFA [3]. Integration of biomarker assessment and imaging such as intracardiac echocardiography (ICE), 3D EAM, ultrasound‐guided venous access in further studies may strengthen insight of procedural safety [4].
3. **Future research suggestions**: The lack of a control group constrains the interpretation about the future clinical value of EAM. Multi‐centered randomized clinical trials analyzing PFA with and without 3D electroanatomical remapping would facilitate determining whether diagnosis and treatment of acute reconnections improves future outcomes.

In conclusion, although this study has inherent limitations, it also provides meaningful and practical insights into integration of PFA with 3D mapping. By highlighting these findings, this study may guide clinicians for broader adoption of multipatient selection forum for investigations. Moreover, these outcomes serve as a foundation for refining patient selection, optimizing procedural strategies, and enhancing efficacy of PFA in clinical practice. Furthermore, the points reported here could encourage further research based on validating these approaches across diverse patient population.

## Funding

The authors have nothing to report.

## Conflicts of Interest

The authors declare no conflicts of interest.

## Data Availability

Data sharing not applicable to this article as no datasets were generated or analyzed during the current study.
